# Determination of Recovery by Total Restitution or Compensation Using Multifrequency Vestibular Tests and Subjective Functional Scales in a Human Model of Vestibular Neuritis

**DOI:** 10.3390/audiolres14060080

**Published:** 2024-11-04

**Authors:** Enrico Armato, Georges Dumas, Flavio Perottino, Matthieu Casteran, Philippe Perrin

**Affiliations:** 1 Research Unit DevAH—Development, Adaptation and Handicap, Faculty of Medicine, University of Lorraine, 54500 Vandoeuvre-lès-Nancy, France; georges.dumas10@outlook.fr (G.D.); matthieu.casteran@univ-lorraine.fr (M.C.); philippe.perrin@univ-lorraine.fr (P.P.); 2 Department of Neurosciences, University of Padova, 35100 Padova, Italy; 3 Department of Oto-Rhino-Laryngology Head and Neck Surgery, University Hospital, 38043 Grenoble, France; 4 Department of Oto-Rhino-Laryngology, Centre Hospitalier des Escartons, 05100 Briançon, France; fperottino@ch-briancon.fr; 5 Faculty of Sport Sciences, University of Lorraine, 54600 Villers-lès-Nancy, France; 6 Research Unit 2LPN—Lorraine Laboratory of Psychology and Neuroscience of Behavioural Dynamics, University of Lorraine, 54000 Nancy, France; 7 Laboratory for the Analysis of Posture, Equilibrium and Motor Function (LAPEM), and Department of Paediatric Oto-Rhino-Laryngology and Head and Neck Surgery, University Hospital of Nancy, 54500 Vandoeuvre-lès-Nancy, France

**Keywords:** vestibular neuritis, recovery by total restitution, recovery by compensation, dizziness handicap inventory, bithermal caloric test, video head impulse test, skull vibration, induced nystagmus test

## Abstract

Background: Vestibular Neuritis (VN) can induce unilateral acute vestibular syndrome (AVS). This study aimed to identify predictive factors of recovery from vestibular neuritis considering total restitution and/or compensation. Methods: In this longitudinal study, 40 patients were included. The initial assessment, performed within 36 to 72 h from the onset (T0), included medical history taking (general and specific), including screening for cardiovascular risk factors (CVRFs), and a battery of diagnostic vestibular tests, comprising the bithermal caloric test (BCT), video head impulse test (VHIT), and skull vibration-induced nystagmus (SVIN) test. All patients also completed a Dizziness Handicap Inventory (DHI). All assessments were repeated 90 ± 15 days later (T3). Subjective compensation criteria were based on the DHI total score, and objective compensation criteria were based on laboratory test results. Four groups of patients (A, B, C, D) were delineated by combining patients with normal vs. abnormal vestibular tests and patients with normal vs. abnormal DHI. Results: CVRFs (but not age or body mass index (BMI)) were associated with a poorer recovery of symptoms. The BCT (lateral semicircular canal paresis %), VHIT (lateral semicircular canal gain), and SVINT (nystagmus slow phase velocity) recovered to normal values in 20%, 20%, and 27% of patients, respectively, at T3. Conclusions: Vascular risk factors (hypercholesterolemia) are correlated with patients who do not recover their symptoms via either total restitution or compensation. There was no significant difference between high- and low-frequency vestibular tests in patients recovering from their symptoms. Some patients with objective recovery may continue to have persistent subjective symptoms.

## 1. Introduction

Vestibular neuritis (VN) is the main cause of so-called acute vestibular syndrome (AVS). It causes long-lasting, continuous vertigo. It is assumed to be of viral origin [1] in relation to possible reactivation of latent herpes virus simplex type 1. However, the most common alternative cause is vascular ischemia. This is a theory based on the recurrent finding that vascular risk factors are more prominent in these patients [2]. The viral hypothesis is supported by the fact that there is radiological evidence of inflammation (either neuronal uptake of gadolinium or aspects of localized partial hypersignal of a vestibular region of the labyrinth with otolithic or semicircular canal enhancement) in the acute phase of vestibular neuritis [3,4,5,6].

Acute symptom onset sometimes makes it difficult to distinguish vestibular neuritis from other clinical pathologies with acute symptoms, as neurovegetative signs are sometimes dominant. Thus, it must be differentiated from ischemic stroke involving the superior cerebellar artery, anterior–inferior cerebellar artery, and posterior–inferior cerebellar artery [7,8]. The diagnosis of vestibular neuritis should be based on clinical history and physical examination, following the HINTS (Head Impulse Nystagmus Test of Skew) protocol [9], which is used to distinguish peripheral from central syndromes.

The lateral and anterior semicircular canals are the most affected in vestibular neuritis (superior vestibular neuritis). In some cases, there can be simultaneous involvement of all three semicircular canals on one side (total vestibular neuritis) [10,11]. However, in a minority of patients, there is selective involvement of the posterior semicircular canal (inferior vestibular neuritis) [12]. Although major vestibular dysfunction is expected in vestibular neuritis, recent studies report some diagnosed cases of “possible vestibular neuritis” with very little, if any, objective deficit seen in vestibular function [13], or selective impairment of the lateral semicircular canal, related to localized labyrinthine damage [12,13,14,15,16]. Several recent papers have reported the presence of normal caloric tests in the context of vestibular neuritis [17,18,19,20,21], suggesting possible isolated “otolithic” forms of vestibular neuritis.

It is well known that after vestibular neuritis, the ipsilateral Vestibulo-Ocular Reflex (VOR) and its pathway are affected. This is documented by the head shaking test (HST), bithermal caloric testing (BCT), rotatory testing, the video head impulse test (VHIT), or Vestibular Evoked Myogenic Potentials (VEMPs). More recently, the skull vibration-induced nystagmus (SVIN) test, which analyses very high frequencies, has been used as a bedside global vestibular test exploring type I inner ear hair cells. SVIN usually shows nystagmus (Ny) beating away from the lesion in vestibular neuritis [22,23].

A vestibular lesion induces changes in the gain and time constant of the VOR pathway [24]. The gain in the VOR is a sign of the intensity of the vestibular response and, therefore, the compensation process. Some restoration and adaptation changes are usually described in these patients, a finding usually interpreted as a “compensatory mechanism” [25].

Patients who suffer from vestibular neuritis can develop persistent long-term disability in their daily activities. Some clinical variables, such as the initial vestibular deficit, an enhanced visual dependency, or the level of anxiety, may, to some extent, predict outcomes in vestibular neuritis patients [26]. Although previous evidence reported that neither caloric paresis nor VOR gain in the VHIT predicted the symptom course in vestibular neuritis [27], other authors suggest that saccadic organization seems to participate in visual target retention and ocular compensation during head impulses in VHIT studies [28,29]. At the present time, measures of residual peripheral vestibular function are a poor measure of functional subjective outcome in vestibular neuritis [30].

The aim of this study was to identify predictive factors of restitution following vestibular neuritis in affected patients by completing the following:

▪ Analyzing and determining possible risk factors of the disease;
▪ Evaluating the compensatory process using changes in perceived disability and in vestibular testing from the onset of symptoms (T0) to 3 months after, i.e., 90 ± 15 days (T3);
▪ Using the results to improve the therapeutic management of vestibular neuritis.

Targeted care is extremely important for patients at risk of developing chronic unsteadiness due to ineffective compensation, as chronic instability can have repercussions on the following levels:

▪ Emotional: because poor compensation can generate insecurity, emotional tension, and panic, which can intensify symptoms in a positive feedback loop;
▪ Social: related to fear of leaving home, which can disrupt social relationships with other people;
▪ Professional: related to deficits in the performance of daily work-related and recreational activities.

## 2. Materials and Methods

### 2.1. Patients

**Figure 1:**
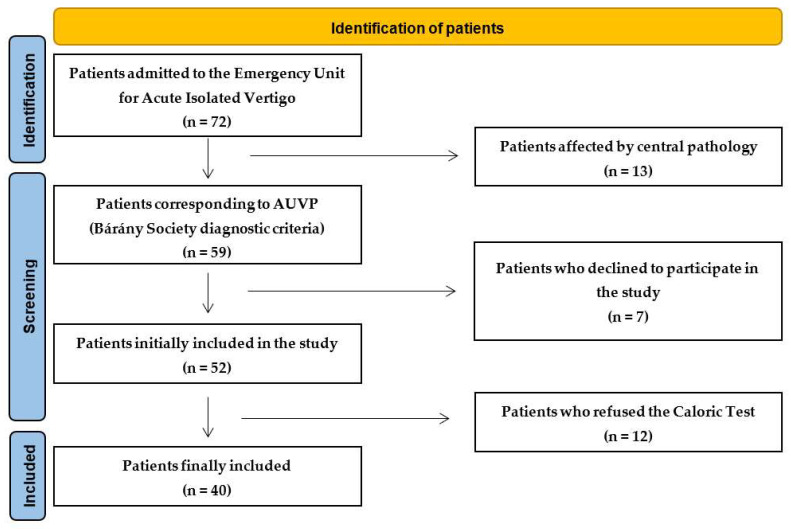
Flow chart diagram according to the PRISMA 2020 guidelines. AUVP: acute unilateral vestibulopathy.

In this longitudinal study, 72 patients admitted to the Emergency Unit from November 2020 to October 2023 because of AVS were referred to the Department of Otorhinolaryngology in the Hospital of Mirano (Venice), Italy. Thirteen patients had documented central pathology confirmed with brain Magnetic Resonance Imaging (MRI). The remaining 59 were diagnosed with vestibular neuritis. Seven patients did not consent to be included in this study; twelve patients did not consent to undergo caloric tests. The remaining 40 patients formed the basis of this study (Figure 1). 

Each patient was assigned a numeric ID, and age, sex, and BMI were collected. Complete anamnesis, as well as neurotological and neurological examinations, were performed in all patients.

Once diagnosed with a peripheral lesion, all patients were encouraged to mobilize as early as possible to achieve a quick return to their daily activities. All patients were advised to perform active dynamic movement protocols at home, several times a day for at least a month.

The inclusion criteria for vestibular neuritis, i.e., acute unilateral vestibulopathy (AUVP), included the following diagnostic criteria established by the Bárány Society [31]:

▪ Acute or subacute onset of sustained spinning or non-spinning vertigo (i.e., acute vestibular syndrome) of moderate to severe intensity with symptoms lasting for at least 24 h;
▪ Spontaneous peripheral vestibular nystagmus, i.e., nystagmus with a direction appropriate to the semicircular canal afferents involved, usually horizontal–torsional, direction-fixed, and enhanced by removal of visual fixation;
▪ Definitive evidence of reduced VOR function on the side opposite to the direction of the fast phase of the spontaneous nystagmus;
▪ No evidence of acute central neurological symptoms or acute audiological symptoms such as hearing loss or tinnitus or other otologic symptoms such as otalgia;
▪ No acute central neurological signs, i.e., no central ocular motor or central vestibular signs; particularly no skew deviation, no gaze-evoked nystagmus, and no acute audiological signs;
▪ Not better accounted for by another disease or disorder.

The non-inclusion criteria concerning our study were as follows:

▪ History of any vestibular or neurotologic disorders (including vestibular migraine);
▪ History of otologic disorders or surgery;
▪ Symptoms (aural fullness, tinnitus, hearing loss) leading to doubt about the initial Menière’s attack or the first recurrent attack of vertigo;
▪ Medical condition or malignancy that can cause an immunocompromised status;
▪ Regular intake of psychotropic drugs;
▪ MRI that revealed acute infarction or other acute/chronic brain lesions, including cerebellopontine angle tumors.

Patients received follow-up assessment three months after the onset of vertigo. All tests necessary to evaluate vestibular impairment and the process of recovery via total restitution or via compensation were performed from the initial assessment to the end of the follow-up.

All patients underwent routinely performed tests only, without invasive or experimental procedures. This study was conducted in accordance with the Declaration of Helsinki and approved by the Ethics Committee of dell’AULSS 3 Serenissima Regione Veneto, Mirano, Venezia, Italy (protocol code 157A/CESC, 22 December 2020). Written informed consent was obtained from all subjects involved in this study.

### 2.2. Methods

#### 2.2.1. Bedside Vestibular Testing

The bedside examination was performed at hospital admission. All patients were treated during the acute phase with tapered doses of glucocorticoid (methylprednisolone starting from 32 mg daily) and antiemetic drugs. The differential diagnosis between peripheral and central vertigo episodes, in the Neuro-otology Department of the first author, was performed clinically by using HINTS plus (HINTS with hearing evaluation) [32], along with monitoring of the symptomatology, with particular attention paid to the severity of imbalance and the duration of acute vertigo. To rule out central lesions, patients who clinically mimicked peripheral AVS syndrome also underwent cerebral MRI.

At the time of the first observation, the medical history of all patients was taken. The anamnesis was general and specific for cardiovascular risk factors (CVRFs), i.e., obesity, arterial hypertension, diabetes mellitus, smoking, and hypercholesterolemia. Eye movement evaluation, performed under videonystagmography (VNG), was related to spontaneous nystagmus and nystagmus evoked by head shaking. The bedside exam was repeated 90 ± 15 days after the first observation. We deliberately chose a “short-term follow-up” to understand the recovery dynamics shortly after the appearance of vestibular neuritis.

#### 2.2.2. Laboratory Vestibular Testing

The laboratory vestibular testing was performed within 36 ÷ 72 h after the onset of symptomatology and was repeated 90 ± 15 days after the first observation. The vestibular system was evaluated at specific response frequencies based on different available diagnostic tests since receptors are frequency-specific [33,34]. In static conditions, type II vestibular receptors or tonic cells have a regular firing rate, which is modulated by very low- and low-frequency head movement and assessed by the BCT. Type I vestibular receptors (phasic cells) with irregular firing rate neurons are activated by high-frequency head movements and assessed by the VHIT and SVINT.

Different types of inner ear hair cells and regular or irregular discharging neurons were detailed by Curthoys et al. [35,36] and by Lacour et al. [37]. It is now accepted that the BCT corresponds to type II inner ear hair cell responses with regular discharging neurons and that SVIN corresponds to the response of type I inner ear hair cells with irregularly discharging neurons [36,38].

Very low vestibular frequencies were evaluated by conventional caloric test (Fitzgerald–Hallpike protocol) (volume: 250 mL; duration: 40 s; temperatures: 30 °C and 44 °C). A water caloric irrigator (Menfis biomedical^®^, Bologna, Italy) was used. A video-based system (Ulmer VNG 2D, ver. C4, Synapsys^®^, Marseille, France) was used for the acquisition and analysis of eye movements. We measured the maximum slow phase velocity (SPV) of the evoked nystagmus for the four irrigations and used the Phillipszoon–Jongkees formula to calculate unilateral weakness and directional preponderance. A unilateral canal paresis exceeding 20% indicated vestibular hypofunction [39].

High vestibular frequencies were evaluated by the VHIT. We used a VHIT Remote Camera System, (Inventis^®^, Padova, Italy). The patient was asked to stare at an earth-fixed target (1.5 cm diameter spot located 1.5m in front), according to the HIMP procedure; then, 20 low-amplitude (10–15°) high-velocity head impulses (200–250°/s horizontal plane; 150–200°/s vertical planes) were randomly administered on each side for all semicircular canals. The correct positioning of vertical canal pairs [left anterior–right posterior (LARP) and right anterior–left posterior (RALP)] on the sagittal plane was always respected. The software automatically calculated the average high-velocity VOR gain, and the asymmetry index was expressed as a percentage (not presented in this study). Normal values were within 15% between the right and left sides. This value resulted from our own data collected from a group of 50 normal subjects ranging from 20 to 80 years of age. The asymmetry index was calculated as GA = (GL − GR)/(GL + GR) × 100, where GA is gain asymmetry, GR is right-sided mean gain, and GL is left-sided mean gain [40]. The asymmetry index reveals differences between the sides in terms of high-velocity VOR [41]. We did not report the asymmetry index measurements obtained with vertical plane stimulation, as in our experience, there is a high level of artifacts [42]. Both covert (early) and overt (late) corrective saccades and the organization of their latencies, in terms of isochrony (gathered) and heterochrony (scattered), were taken into consideration [43]. Both the corrective saccades and VHIT gain detect vestibular hypofunction [31].

Very high vestibular frequencies were evaluated by the SVINT or Dumas’ Test. A 100 Hz vibrator (VVIB Synapsys^®^, Marseille, France) was applied for 5 to 10 s on the right/left mastoid and vertex site; the slow phase velocity of the evoked nystagmus was recorded by an infrared camera (Ulmer VNG 2D system, ver. C4, Synapsys^®^, Marseille, France). The test was considered negative if there was an absence of nystagmus, a slow phase velocity lower than 2.5°/s, or a lack of concordance of the direction of nystagmus on the two mastoids [38,44].

**Figure 2:**
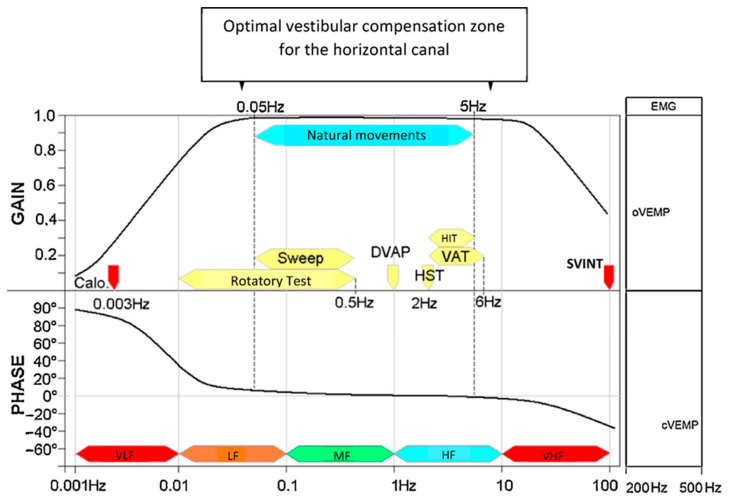
The currently known frequency spectrum of the vestibular system. Graph summarizing the complementarity of vestibular tests, sorted by stimulation frequency. The bithermal caloric test (BCT) is representative of very low frequencies, the video head impulse test (VHIT) of high frequencies, and the skull vibration-induced nystagmus test (SVINT) of very high frequencies (VLFs: very low frequencies; LFs: low frequencies; MFs: middle frequencies; HFs: high frequencies; VHFs: very high frequencies; Calo: caloric test; Sweep: multifrequency sinusoidal test; DVAP: Dynamic Visual Acuity Performance; HST: head shaking test; VAT: vestibular autorotation test; HIT: head impulse test; EMG: electromyography; oVEMP: ocular vestibular evoked myogenic potentials; cVEMP: cervical vestibular evoked myogenic potentials) [44,45,46].

Measuring vestibular alterations in vestibular neuritis usually requires separate explorations of three semicircular canals or the otoliths. It has recently been shown that the SVINT provides a more global vestibular test, as it assesses the three semicircular canals (mainly the lateral and superior ones) but also the utricle. The horizontal component of the SVINT is capable of measuring both lateral semicircular canal and utricular responses [44,45,46] (Figure 2).

#### 2.2.3. Questionnaires

The questionnaire was the validated Italian version of the Dizziness Handicap Inventory (DHI) [47,48]. It was performed at hospital admission and repeated 90 ± 15 days after the first observation.

The DHI is a patient-reported outcome measure designed to measure the disability perceived by someone with complaints of dizziness, vertigo, or unsteadiness. The total score documents changes related to functional, emotional, and physical domains [49].

In the literature, there are no common criteria on the severity scale of the DHI. Many authors adopt the following: (a) mild handicap; a score of 0–30; (b) moderate handicap; a score of 31–60; and (c) severe handicap; a score of 61–100 [49,50,51,52]. Another method of reporting is the following: (a) asymptomatic, fully recovered (DHI = 0); (b) low symptoms (DHI 2–28); and (c) high symptoms (DHI 36–80) [53]. We adopted the suggestion in the literature and established an arbitrary criterion of 32 points in the DHI to determine the state of general vestibular compensation in the patients. Those with a DHI total score < 32 were reported as “compensated,” and those with a DHI total score ≥ 32 were reported as “non-compensated” [54].

### 2.3. Clinical Course of Vestibular Neuritis

The clinical course of vestibular neuritis can be described as follows:

▪ Total restitution: complete recovery of functional activity of the labyrinth sometimes occurs, due to the resolution of the lesion and restoration of normal vestibular function through cellular regeneration and the disappearance of edema [55].
▪ Compensation: there is occasionally an incomplete recovery of the functional activity of the labyrinth. In this case, a recalibration process of the activity of the vestibular nuclei must take place. This is enabled by a high degree of plasticity and occurs in the central nervous system in response to the sensory deficit [35,37]. The goal of the compensation process is to re-establish symmetrical neuronal activity at the level of the vestibular nuclei [36].

To evaluate the compensation status of our patients, we utilized the work of Guajardo-Vergara et al. [54] based on the ideas of Eisenman et al. [56]. There are slight differences in the cut-off values of objective tests and the proposal of subjective scales (for more details, see Section 4.1, which provides a summary of vestibular compensation criteria in the literature).

One concern was that the proposed classification from Guajardo-Vergara et al. [54] does not take into account the situation of patients with normalized vestibular assessments who still have persistent dizzy symptoms as evaluated by the Perez-Rey (PR) index. Patients with persistent postural–perceptual dizziness (PPPD), i.e., a functional neuro-otologic disorder that is the most frequent cause of a chronic vestibular syndrome (dizziness, unsteadiness, and non-spinning vertigo, exacerbated by an upright posture or walking, active or passive motion, and exposure to moving or complex visual stimuli) [57] fall into this category.

We utilized bedside and laboratory test results (measuring objective recovery and objective compensation) and combined them with the DHI total score (measuring subjective symptoms) to define four groups (A, B, C, D) of patients (Table 1):

Development via *total restitution* requires the normalization of all clinical and laboratory tests, but it can be associated with different DHI total scores.

▪ A group: DHI score < 32: complete recovery, as measured by clinical and laboratory tests and no subjective symptoms, i.e., total restitution.
▪ B group: DHI total score ≥ 32: complete recovery, as measured by clinical and laboratory tests, with some persistent subjective symptoms.

Development via *compensation* in patients with persisting abnormal vestibular clinical and laboratory tests must be divided into two subgroups, depending on whether compensation has been *ineffective* (“uncompensated”) or *effective* (“well compensated”).

▪ C group: the objective vestibular test recovery and compensation criteria [54] are not met (uncompensated) and are associated with subjective symptoms, i.e., a DHI total score ≥ 32.
▪ D group: the objective vestibular tests remain modified to differing degrees, and the compensation criteria [54] are met (compensated) and are not associated with subjective symptoms, i.e., a DHI total score < 32.

### 2.4. Patient Categories According to Clinical and Vestibular Results 

We initially divided our group of 40 patients based on the DHI score, allowing the creation of two group pairs, i.e., DHI < 32 (A + D group) vs. DHI ≥ 32 (B + C group). The A + D group represents patients who have recovered with no reported symptoms. The B + C group represents patients who are still impaired by symptoms in their daily lives. 

A second group pair was created based on patients’ objective status, allowing the creation of two further group pairs, i.e., with normal laboratory tests (A + B group) vs. patients with objective deficits measured by vestibular tests (C + D group) [for more references, see Section 3.3.2, which reports our data on the comparison of two group pairs].

### 2.5. Statistical Analysis

The parameters examined were age, sex, BMI, CVRFs (arterial hypertension, diabetes mellitus, hypercholesterolemia), and the results of the DHI, BCT, VHIT, and SVINT.

All statistical analyses were performed using Jamovi software v2.3.28 (Jamovi project (2022)). Age, sex, and BMI were analyzed between the A + D group and the B + C group, as well as the A + B group and the C + D group. We also performed a binomial logistic regression to identify the best contribution of each feature. We also looked at differences between T0 and T3 between the A + D group and the B + C group, as well as for the A + B group and the C + D group.

A categorical variable (sex) was compared using a Fisher exact test for the A + D group and the B + C group and for the A + B group and the C + D group. For continuous variables (BMI and age), a normal distribution was verified using the Shapiro–Wilk test, and the homogeneity of variances was verified with Levene’s test. Both conditions were met; thus, we performed a Student’s t-test. In each case, our hypothesis was that both groups were different.

We also carried out a binomial stepwise backward logistic regression analysis to determine the optimal combination of features that separates the two groups, i.e., between patients with no symptoms and patients with symptoms or between patients with normal test results and patients with abnormal results. Hypertension, diabetes, hypercholesterolemia, smoking, and physiotherapy treatment were used as input variables. BMI was used as a covariable. The variance indication factor (VIF) is a measure of the amount of multicollinearity in a set of multiple regression variables. A high VIF indicates that the variable is redundant or strongly related to other predictors. A combination of variables was chosen to yield the highest sensitivity and specificity. The outcome of the best classification matrix (i.e., with the highest sensitivity and specificity) should be equal to 1. A binomial logistic regression was performed to assess the influence of each parameter in the different groups (i.e., which parameters were contributing maximally). A sensitivity and a specificity of 100% would be expected for a combination of features predicting a perfect classification.

A normal distribution was also verified for the BCT, VHIT, and SVINT data using the Shapiro–Wilk test, and the homogeneity of variances was verified with Levene’s test. When both conditions were met, parametric tests were performed. In the case of non-compliance with the homogeneity of variances, a Welch’s t-test was conducted. In the case of non-compliance with the normality of the distribution, non-parametric tests were used. For group comparisons (A + D vs. B + C or A + B vs. C + D at T0 or T3), tests for independent samples were implemented (Student’s t-test or Wilcoxon rank-sum test). For before-and-after comparisons within the same group (A + D between T0 and T3, likewise for A + B, B + C, and C + D), paired samples tests were performed (Mann–Whitney U test or Welch’s t-test). In each case, the hypothesis was that one group would be superior to another and not just different. 

For all statistical analyses, the level of significance was set at *p* = 0.05. Significance was expressed as follows * = *p* < 0.05; ** = *p* < 0.01; *** = *p* < 0.001; ns = *p* > 0.05.

## 3. Results

### 3.1. Sociodemographic and Clinical Features

The sample consisted of 40 patients, including 21 males (52.5%) and 19 females (47.5%) (i.e., an M/F ratio of 1.10/1). The age was 55.91 ± 13.9, and the BMI was 25.71 ± 3.54 (normal values: 18.5 ÷ 25). The time between the onset of symptoms and the first evaluation was 3.25 ± 1.62 days. The time interval between the first and second clinical and laboratory evaluations and the questionnaires was 96.65 ± 6.61 days. Vestibular neuritis affected the right side in 25 patients (62.5%) and the left side in 15 patients (37.5%). At the onset of vestibular neuritis, 16 patients (40%) had reported arterial hypertension treated by regular therapy, 6 patients (15%) had diabetes mellitus, 14 patients (35%) had hypercholesterolemia, and 8 patients (20%) were smokers. Nineteen patients (47.5%) did not perform the proposed vestibular rehabilitation exercises after diagnosis. 

### 3.2. Definition of Patient Groups

Four groups were delineated following questionnaires and vestibular exploration results. The Fisher exact test between patients with DHI < 32, (A + D group) and patients with DHI ≥ 32 (B + C group) was *p* > 0.001. Similarly, the Fisher exact test between patients without vestibular laboratory test dysfunction (A + B group) and patients with vestibular laboratory test dysfunction (C + D group) was *p* > 0.001 (Table 1a,b).

### 3.3. Results for Considered Parameters and Groups

#### 3.3.1. Results for the 40-Patient Group

Table 1b shows the global evolution between T0 and T3 for the different groups (A, B, C, D) considering the results of the subjective scale DHI and of objective vestibular tests (BCT, VHIT, SVINT, HST), in terms of the positive or normalized test.

For groups A, B, C, and D (40 patients), the normalization of objective tests from T0 to T3 was 20%, 20%, and 27% for the BCT, lateral semicircular canal VHIT, and SVIN, respectively, and 80% for the HST.

For the A + D group (patients without symptoms or DHI < 32), the results concerning normalization of objective tests from T0 to T3 were 24%, 24%, and 31% for the BCT, lateral semicircular canal VHIT, and SVIN, respectively, and 100% for the HST.

For the B + C group (patients with symptoms or DHI ≥ 32), the results concerning the normalization of objective tests from T0 to T3 were 9%, 9%, and 9% for the BCT, lateral semicircular canal VHIT, and SVIN, respectively, and 75 % for the HST. 

The A + D group (patients without symptoms or DHI < 32) showed low percentages of recovery, except for the anterior semicircular VHIT gain.

The B + C group (patients with symptoms or DHI ≥ 32) showed very low percentages of recovery for all the vestibular laboratory tests.

The A + B group (patients with no vestibular laboratory test dysfunction) reached 100% recovery in nearly all vestibular laboratory tests.

The C + D group (patients with vestibular laboratory test dysfunction) showed either no recovery or very low amounts, except for the anterior semicircular VHIT gain (Table 2).

**Figure 3:**
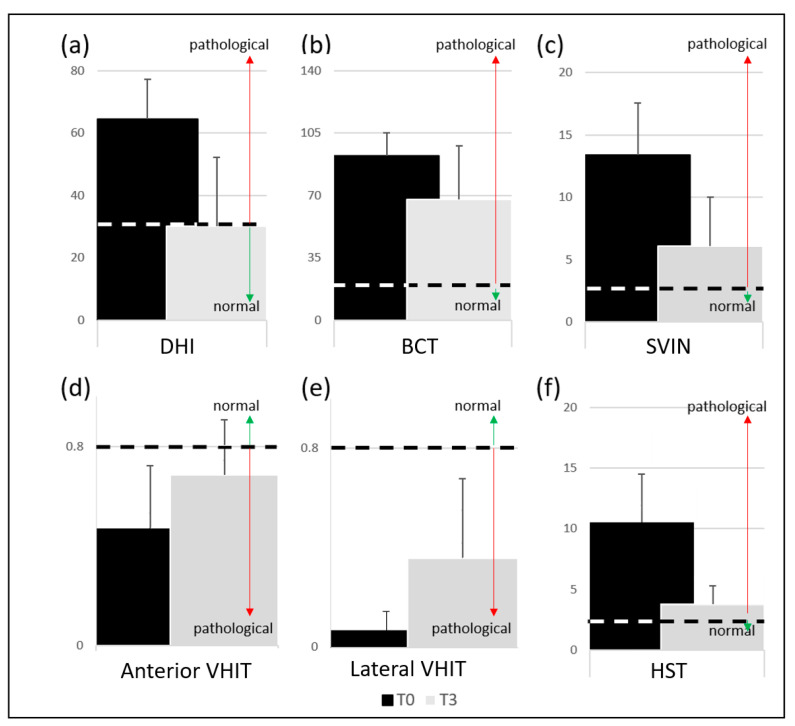
Graphical representation by means and standard deviations for the 40-patients group at T0 (black) and T3—after 3 months (grey) for (**a**) Dizziness Handicap Inventory (y-axis = total score); (**b**) bithermal caloric test (y-axis = percentage of canal paresis); (**c**) skull vibration-induced nystagmus test (y-axis = nystagmus slow phase velocity); (**d**) anterior semicircular canal video head impulse test (y-axis = video head impulse test gain); (**e**) lateral semicircular canal video head impulse test (y-axis = video head impulse test gain); and (**f**) the head shaking test (y-axis = nystagmus slow phase velocity). The dashed lines represent the boundary between normal and pathological values. The red arrow represents the extent of pathological values, and the green arrow represents the extent of normal values. Dizziness Handicap Inventory (DHI), bithermal caloric test (BCT), skull vibration-induced nystagmus test (SVINT), head impulse test (VHIT), head shaking test (HST).

The 40-patient group comes from the sum of the A + B + C + D groups. By comparing the percentage of recovery between T0 and T3 for the questionnaire and laboratory tests, we found the following recovery percentages: DHI 72.5%, BCT 20%, lateral semicircular canal VHIT 20%, anterior semicircular canal VHIT 55%, SVINT 27%, and HST 80%. Figure 3 shows the DHI total scores (a), BCT percentage of canal paresis (b), SVIN nystagmus slow phase velocity (c), lateral (d) and anterior (e) semicircular canal VHIT gain, and HST slow phase nystagmus (f) at T0 and T3. 

#### 3.3.2. Results by Comparison of the Four Paired Groups

- ***Age, Sex, and BMI*** for the A + D group vs. the B + C group (DHI < 32 vs. DHI ≥ 32).

No differences were found between the two pairs of groups. These parameters do not contribute to distinguishing groups of patients who complain or do not complain of subjective symptoms (Table 3).

- ***Age, Sex, and BMI*** for the A + B group vs. the C + D group (normal exams vs. pathological exams).

No differences were found between the two pairs of groups. These parameters do not contribute to distinguishing groups of patients who regularize or do not regularize their objective vestibular tests (Table 3), as for any factor, *p* is >0.05, i.e., NS.

- ***Cardiovascular risk factors*** for the A + D group vs. the B + C group (DHI < 32 vs. DHI ≥ 32).

The logistic regression analysis defined diabetes and hypercholesterolemia as indicators for less recovery, with a 54.5% sensitivity and a 96.6% specificity from the classification matrix. VIFs were equal to 1.02 for diabetes and hypercholesterolemia.

- ***Cardiovascular risk factors*** for the A + B group vs. the C + D group (normal exams vs. pathological exams).

The logistic regression analysis defined hypertension, diabetes, and hypercholesterolemia as indicators, with a 96.9% sensitivity and a 62.5% specificity from the classification matrix. The recovery of vestibular objective function (vestibular tests) is less favorable for patients with hypercholesterolemia. VIFs were equal to 1.69 for diabetes and hypertension and equal to 1.01 for hypercholesterolemia.

- ***BCT*** for the A + D group vs. the B + C group (DHI < 32 vs. DHI ≥ 32).

**Figure 4:**
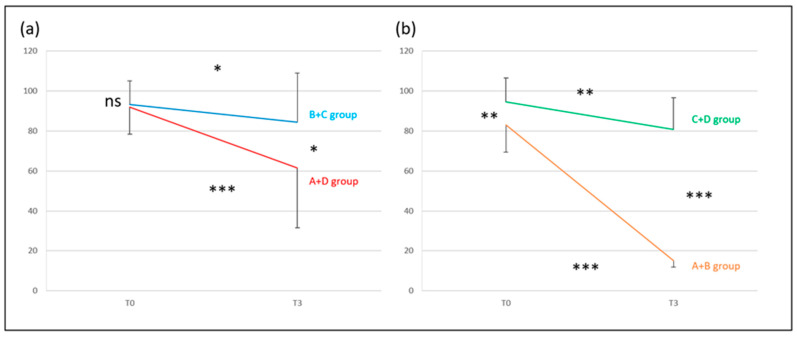
Bithermal caloric test: Lateral semicircular canal paresis percentage (y-axis = percentage of canal paresis) at T0 and T3. (**a**) A + D (patients without symptoms at T3, red line) and B + C (patients with symptoms at T3, blue line) groups; (**b**) A + B (patients with no vestibular laboratory test dysfunction at T3, orange line), and C + D (patients with vestibular laboratory test dysfunction at T3, green line) groups. The last result suggests that when the initial BCT at T0 shows a hypofunction > 90%, the recovery of caloric function is very poor, and the lesion is permanent. Conversely, for a BTC hypofunction < 80% at T0, recovery is possible at T3 and can be complete in some cases. Significance is expressed as follows: * = *p* < 0.05; ** = *p* < 0.01; *** = *p* < 0.001; and ns = *p* > 0.05.

At T0, there was no difference between the two pairs of groups (NS), but there was a difference at T3 (*p* < 0.05). The recovery of the B + C group was not significant (*p* < 0.05). Conversely, there was a significant recovery for the A + D group (*p* < 0.001); however, the recovery was still partial and incomplete, with no complete return to normal. At T3, 24% of the initial T0 abnormal values recovered normal function in the A + D group and 9% for the B + C group (Figure 4a).

- ***BCT*** for the A + B group vs. the C + D group (normal exams vs. pathological exams).

The groups were different at T0 (*p* < 0.01). The groups were more different at T3 (*p* < 0.001), as the recovery of the A + B group was significantly higher than that of the C + D group. The recovery of the A + B group was significant (*p* < 0.001), with a return to normal. On the contrary, there was a significant recovery for the C + D group (*p* < 0.01), with no complete return to normal. At T3, 100% of the initial T0 abnormal values recovered normal function in the A + B group and 0% in the C + D group (Figure 4b).

- ***VHIT*** for the A + D group vs. the B + C group (DHI < 32 vs. DHI ≥ 32).

**Figure 5:**
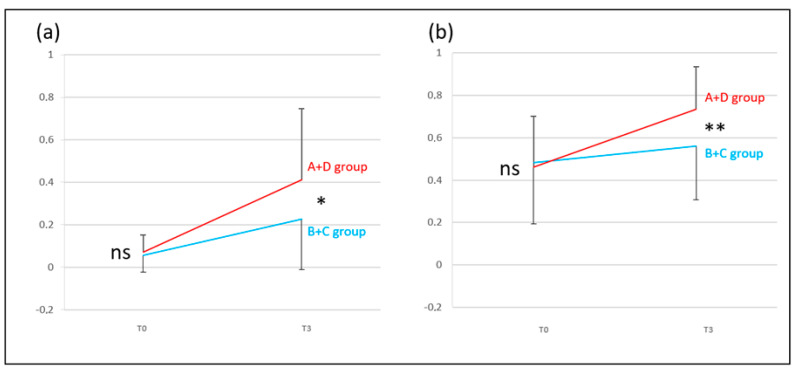
Video head impulse test gain averages for left and right vestibular neuritis at T0 and T3 (y-axis = video head impulse test gain) for the A + D (patients without symptoms, red line) and B + C (patients with symptoms, blue line) groups. (**a**) lateral semicircular canal and (**b**) anterior semicircular canal. Significance is expressed as follows: * = *p* < 0.05; ** = *p* < 0.01; and ns = *p* > 0.05.

The A + D group showed a difference compared with the B + C group (difference T0 and T3) for the lateral and anterior semicircular canals (*p* < 0.05 and *p* < 0.01). The A + D group had a better development of the VHIT gain level than the B + C group between T0 and T3 for the lateral and anterior semicircular canals. For the lateral semicircular canal at T3, 24% of the initial T0 abnormal values recovered normal function for the A + D group and 9% for the B + C group. For the anterior semicircular canal at T3, 59% of the initial T0 abnormal values recovered normal function for the A + D group and 36% for the B + C group (Figure 5).

- ***VHIT*** for the A + B group vs. the C + D group (normal exams vs. pathological exams).

**Figure 6:**
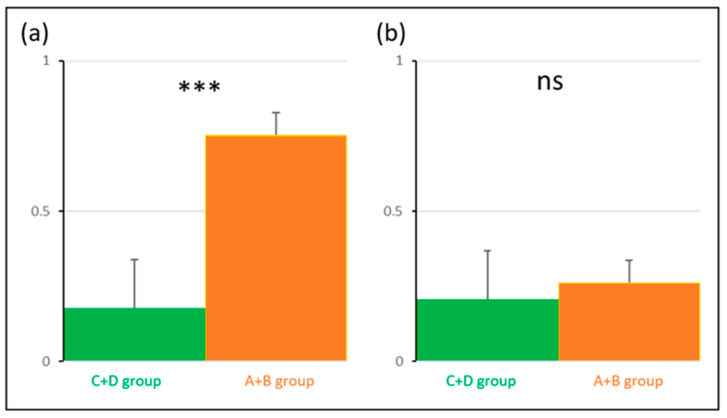
Evolution of video head impulse test gain averages (y-axis = video head impulse test gain) on the affected side for left and right vestibular neuritis at T0 and T3 for the A + B (patients with no vestibular laboratory test dysfunction, orange plot) and C + D (patients with vestibular laboratory test dysfunction, green plot) groups. (**a**) lateral semicircular canal and (**b**) anterior semicircular canal. Significance is expressed as follows:; *** = *p* < 0.001; and ns = *p* > 0.05.

The A + B group showed a difference compared with the C + D group (difference T0 and T3) only for the lateral semicircular canal (*p* <0.001). These results showed that the A + B group had a better change in level than the C + D group between T0 and T3 for the lateral semicircular canal. There was no change in the A + B and C + D group for the anterior canal. For the lateral semicircular canal at T3, 100% of the initial T0 abnormal values recovered normal function for the A + B group and 0% for the C + D group. For the anterior semicircular canal at T3, 100% of the initial T0 abnormal values recovered normal function for the A + B group and 44% for the C + D group (Figure 6).

- ***SVINT*** for the A + D group vs. the B + C group (DHI < 32 vs. DHI ≥ 32).

**Figure 7:**
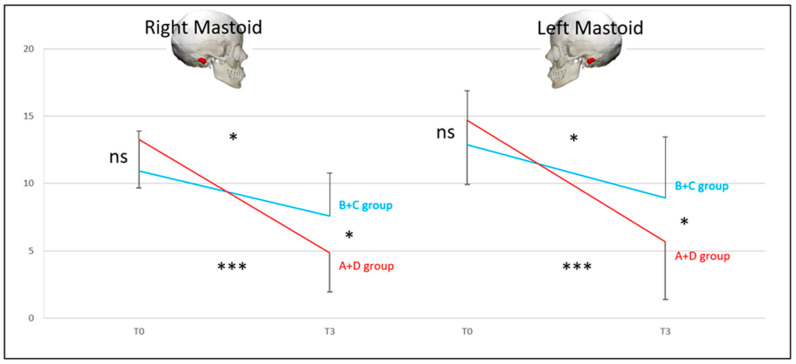
Skull vibration-induced nystagmus slow phase velocity averages (y-axis = skull vibration-induced nystagmus slow phase velocity) for the right and left mastoids at T0 and T3 for the A + D (patients without symptoms, red line) and B + C (patients with symptoms, blue line) groups. Significance is expressed as follows: * = *p* < 0.05; *** = *p* < 0.001; and ns = *p* > 0.05.

At T0, there was no difference between the two pairs of groups (ns). The A + D (*p* < 0.001) and B + C (*p* < 0.05) groups were different at T3 compared with T0 for the SVINT on stimulating the right and left mastoid. For the right mastoid stimulation at T3, 34% of the initial T0 abnormal values recovered normal function for the A + D group and 9% for the B + C group. For the left mastoid stimulation at T3, 34% of the initial T0 abnormal values recovered normal function for the A + D group and 18% for the B + C group. At T3, there was a significant difference between the two pairs of groups (*p* < 0.05) for right and left mastoid. There were no significant differences between the right and left mastoid (Figure 7).

- ***SVINT*** for the A + B group vs. the C + D group (normal exams vs. pathological exams).

**Figure 8:**
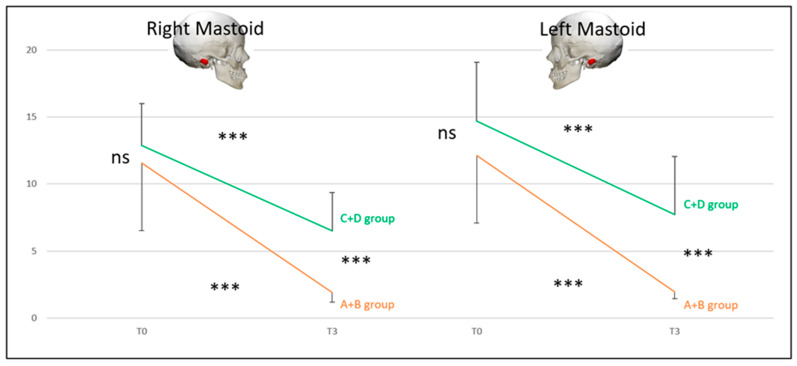
Comparison of skull vibration-induced nystagmus slow phase velocity averages (y-axis = skull vibration-induced nystagmus slow phase velocity) for the right and left mastoids at T0 and T3 for the A + B (patients with no vestibular laboratory test dysfunction, orange line) and C + D groups (patients with vestibular laboratory test dysfunction, green line) groups. Significance is expressed as follows; *** = *p* < 0.001; and ns = *p* > 0.05.

At T0, there was no difference between the two pairs of groups (NS), but there was a difference at T3 (*p* < 0.001). The C + D and A + B groups were different at T3 compared with T0 (*p* < 0.001). For the right mastoid stimulation at T3, 87.5% of the initial T0 abnormal values recovered normal function for the A + B group and 12.5% for the C + D group. For the left mastoid stimulation at T3, 100% of the initial T0 abnormal values recovered normal function for the A + B group and 12.5% for the C + D group. There were no significant differences between the right and left mastoids (Figure 8).

## 4. Discussion

The major findings of our study showed that CVRFs were associated with poorer symptom recovery; conversely, neither age nor BMI were correlated with recovery. On vestibular testing, 20% of the patients recovered to normal at T3 as measured by the BCT; for the lateral semicircular canal VHIT, the value was also 20%, and for the SVINT, there was 27% recovery.

To clarify these results, a group corresponding to vestibular neuritis patients with normal vestibular laboratory tests and persistent subjective symptoms was proposed (B group), related to patients we regularly see in our clinic with symptoms of PPPD after vestibular neuritis.

### 4.1. Vestibular Compensation Criteria in the Literature

In 2001, Eisenman et al. [56] stated that compensation can be considered incomplete when patients present any one of the four proposed criteria. In 2020, Guajardo-Vergara et al. [54] partially modified these criteria. 

In contrast to the original classification proposed by Eiseman et al. [56], Guajardo-Vergara et al. [54] considered each criterion in isolation. They decided to add the DHI total score, and following previous data, they established an arbitrary criterion of 32 points in the DHI to determine the state of general vestibular compensation of the subjects. Those with a DHI total score < 32 were described as compensated and those with a DHI total score ≥ 32 were non-compensated (Table 4).

Guajardo-Vergara et al. [54] also showed how the Perez-Rey index, measured by the v-HIT, could allow for the separation of a compensated group from a non-compensated group. They utilized the DHI assessment and rotatory chair results to categorize a Perez-Rey index cut-off value of 55 to distinguish the two groups. This measure also has the potential to serve as a criterion in the follow-up evaluation of patients suffering ongoing symptoms after an AVS.

The Perez-Rey index diminishes in parallel (1) with the reduction in the DHI score in patients with chronic instability because of a long-standing unilateral vestibulopathy and (2) after a vestibular rehabilitation program [58]. These findings indicate that saccades in different impulses tend to appear time-locked from the initiation of the head movement onward, whereas, in non-compensated patients, the Perez-Rey index remains high [28,46].

When vestibular compensation takes place, overt corrective saccades progressively decrease in number and velocity and covert corrective saccades transform from scattered to gathered, although the VOR gain remains unchanged [58].

The corrective saccades type and the time of appearance can provide valuable information on the level of vestibular neuritis compensation: the presence of only covert saccades is associated with a higher degree of compensation, while the simultaneous presence of covert and overt saccades is associated with a lower degree of compensation [50].

We based our study on a questionnaire (DHI) to evaluate subjective symptoms and three laboratory tests (BCT, VHIT, SVINT), covering the vestibular system bandwidth, to evaluate the objective level of total restitution and compensation. We adopted the Guajardo-Vergara et al. compensation criteria, especially the cut-off for the DHI total score (≥32 for pathologic patients).

**Figure 9:**
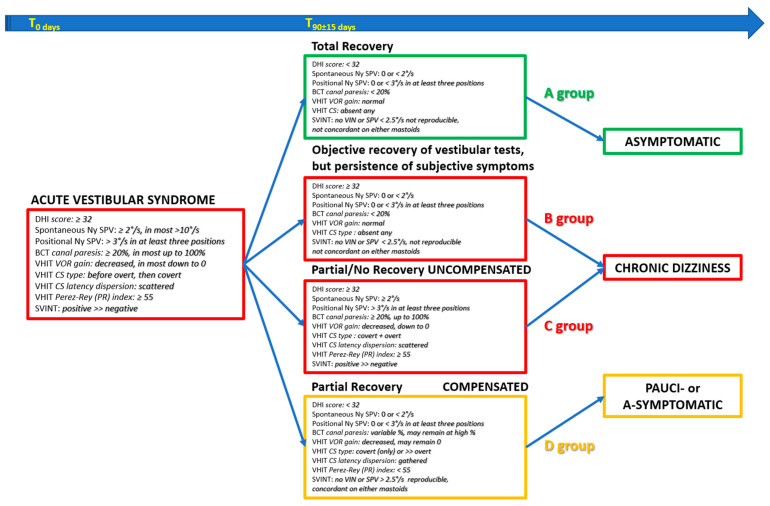
Vestibular neuritis evolution diagram. On the left: The onset of vestibular neuritis is parameterized through Dizziness Handicap Inventory (DHI), spontaneous nystagmus (Ny), bithermal caloric test (BCT), video head impulse test (VHIT), and skull vibration induced nystagmus test (SVINT) (tests are ordered by labyrinthine analysis frequency from lowest to highest). In the center: Development of vestibular neuritis from the normalization of all tests with normal (A group) or pathological (B group) DHI to the persistence of pathological tests with ineffective objective laboratory test compensation parameters and pathological DHI (C group) or pathological tests with effective objective laboratory test compensation parameters and normal DHI (D group). On the right: Symptom profile of the various evolutive groups, i.e., asymptomatic (A group), chronic dizziness (B and C groups), and pauci- or asymptomatic (D group). Slow phase velocity (SPV), vestibular oculomotor reflex (VOR), corrective saccades (CS), vibration induced nystagmus (VIN).

A summary diagram is proposed (Figure 9). It shows the parameters (DHI, spontaneous nystagmus, BCT, VHIT, and SVINT) that characterize vestibular neuritis at its onset. Based on the development of the parameters considered, four lines are considered, which correspond to the proposed groups: normalization of all tests with normal (A group) or pathological (B group) DHI, persistence of pathological tests with ineffective objective laboratory test compensation parameters and pathological DHI (C group), and pathological tests with effective objective laboratory test compensation parameters and normal DHI (D group). The symptom profiles of the various groups are as follows: asymptomatic (A group); chronic dizziness (B and C groups); and minimal, if any, symptoms (D group).

### 4.2. Cardiovascular Risk Factors Among Patients with Vestibular Neuritis

A significantly higher prevalence of CVRFs was found among patients presenting with vestibular neuritis in comparison with the general population [59]. This finding may suggest a possible correlation between CVRFs and vestibular neuritis, which may be mediated through small blood vessel occlusion and labyrinthine ischemia.

Three-dimensional high-resolution temporal bone histopathology has shown evidence that the anatomy of the labyrinth may make the superior vestibular artery more vulnerable to external compression if the adjacent superior vestibular nerve becomes edematous in response to injury or inflammation or is affected by a tumor. While the veins do not appear to be vulnerable to adjacent neural edema, an arteriovenous crossing was found in both high-resolution images where the inferior vestibular artery crosses the superior vestibular vein. In this way, viral damage to the nerve could also be mediated by a vascular mechanism [60].

Another study did not observe a significant difference in the prevalence of arterial hypertension, diabetes mellitus, dyslipidemia, prior history of stroke, transitory ischemic attack, or active smoking between patients with idiopathic benign paroxysmal positional vertigo (BPPV) and BPPV secondary to peripheral AVS. This means that clinically, it is not possible to determine whether AUVP is due to ischemia of the anterior vestibular artery or inflammation of the superior vestibular nerve [61].

A recent investigation showed that patients with superior vestibular neuritis did not present with more CVRFs (age, sex, high blood pressure, hypercholesterolemia, diabetes, smoking, documented cardiovascular disease, and atrial fibrillation) than the general population. Hypercholesterolemia was defined as LDL-cholesterol > 1.6 g/L or ongoing lipid-lowering treatment. The study argues for the involvement of the superior vestibular nerve rather than the anterior vestibular artery in superior vestibular neuritis [62].

Finally, a retrospective cohort study stated that patients with vestibular neuritis and CVRFs were older on average than patients without CVRFs; however, there were no significant differences in the mean VOR gain at diagnosis or follow-up between the groups with and without CVRFs. Factors such as age and CVRFs did not significantly impact the VOR gain at diagnosis [63].

In our study, diabetes and hypercholesterolemia were factors associated with persistent subjective complaints (B + C group). In the same way, hypercholesterolemia played an important role in patients who did not recover instrumentally (C + D group).

In patients examined during the acute phase of vestibular neuritis, it may be useful to measure cholesterol. In the case of increased levels, statin therapy should be administered.

### 4.3. Development of Laboratory Testing in Vestibular Neuritis

Our results showed that all vestibular tests including semicircular canal explorations (BCT, VHIT) and more global tests including semicircular canals and utricle function (SVINT) improved in a similar fashion, regardless of the frequency assessed. Test results also showed that there was no strict relationship with subjective recovery.

#### 4.3.1. Bithermal Caloric Test (BCT)

In the literature, there is a debate on (1) whether or not canal paresis can improve over time and (2) whether or not there is a relationship between severity and patient outcomes.

Regarding the development of canal paresis, lateral semicircular canal impairment usually persists for more than a year in vestibular neuritis [17,19,28,64,65,66]. One other study showed a normalization of the canal paresis in 35% of patients one year after symptom onset [67], and another study with long-term follow-up showed a complete recovery of labyrinthine function, assessed by caloric irrigation, in 50 to 70% of cases [68,69]. The low-frequency function of the labyrinths often recovered its symmetry [12,70]. In summary, there are conflicting opinions regarding the incidence of improvement in canal paresis.

Regarding any correlation between symptoms and canal paresis, it has been shown that there is no relationship between the BCT and the disappearance of symptoms, as patients who are symptom-free can still have a persistent reduction in caloric responses [27,71,72,73]. In our study, there was no correlation between the degree of persistent canal paresis and residual symptoms.

In our group of 40 patients, 20% recovered normal caloric function between T0 and T3. The groups with persistent subjective complaints (B + C) and the symptom-free groups (A + D) both showed abnormal canal paresis at T3, but this was worse in the B + C group.

#### 4.3.2. Video Head Impulse Test (VHIT)

In the literature, there is debate about (1) whether or not VHIT gain improves after the acute phase, (2) whether or not there is a correlation between outcome and VHIT gain, and (3) whether or not there is a correlation between outcome and the appearance and organization of VHIT corrective saccades.

Regarding VHIT gain improvement, the high-frequency response often remains impaired [70]. Although total recovery can take place, in some cases, there is no improvement [12,42,50,74,75,76,77]. Peripheral recovery or central increase in VOR gain on the affected side may explain the improvement in VHIT gains [28,37,39,54,64,65,69,78,79,80,81]. In summary, there are conflicting views regarding the incidence of improved VOR gain (no improvement, partial improvement, or total improvement).

Regarding any correlation between symptoms and VHIT gain, there is no relationship between the lateral semicircular canal VOR gain and chronic symptoms following vestibular neuritis [27,50,54,66,70,72]. Another study suggested that an increase in VOR gain can predict an improvement in symptoms [77]. Patients with abnormal VHIT gain continue to be troubled by many chronic symptoms [66,75,76,81]. In summary, there are conflicting opinions regarding the correlation between VOR gain and residual symptoms.

Regarding any correlation between symptoms and VHIT corrective saccades, patients with mixed covert and overt saccades continue to be troubled by many chronic symptoms. Covert saccades can play a key role in compensation for inadequate VOR response and return to a normal lifestyle [28,37,50,66,75,76,77]. In summary, the appearance of covert saccades only (with no overt saccades seen), with a similar latency (high Perez-Rey index) may play a role in compensating for a functionally inadequate VOR.

In our study of 40 patients, 20% recovered the lateral semicircular canal response between T0 and T3, and 55% recovered the anterior semicircular canal response between T0 and T3.

For lateral semicircular canal responses at T3, 24% of the initial T0 abnormal values recovered normal function for the A + D group and 9% for the B + C group. For anterior semicircular canal responses at T3, 59% of the initial T0 abnormal values recovered normal function for the A + D group and 36% for the B + C group. There was no change in the A + B and C + D groups for anterior canal responses. A higher resistance to injury and the higher recovery possibilities of the anterior semicircular canal compared with the lateral semicircular canal have been reported in the literature [41].

#### 4.3.3. Skull Vibration-Induced Nystagmus Test (SVINT)

In the literature, there is agreement about SVIN not being modified by vestibular compensation in the case of persistent lesions on type I inner ear hair cells and/or irregular discharging neurons. In this situation, the SVINT reveals a persistent asymmetry in activity after stimulation at the level of the vestibular nucleus [36,38,45,82].

There is a positive linear correlation between VOR gain asymmetry and the SVIN slow phase velocity at 100 Hz. This finding adds more knowledge about SVIN: an asymmetry in vestibular function measured with the VHIT leads to a positive SVIN test [22].

The SVIN slow phase velocity, VOR gain asymmetry, and Perez-Rey index decrease over time after unilateral vestibular loss. In addition, there is a positive association between the SVIN slow phase velocity and VOR gain difference, as well as between the SVIN slow phase velocity and the Perez-Rey index. Both tests are useful in the follow-up of vestibular neuritis, as they could reflect clinical compensation, or, more accurately, partial recovery for type I inner ear hair cells or fibers with irregular discharges [23].

SVIN was confirmed in our study to be a pertinent and sensitive test since it was positive in all cases of vestibular neuritis.

In our study, 27% of our patients recovered between T0 and T3. Skull vibration-induced nystagmus was slightly reduced at T3 in patients with persistent subjective complaints (B + C group).

### 4.4. Patients with Increased DHI Total Score and Normal Laboratory Tests

In our study, a possible occurrence of PPPD during vestibular neuritis development was only observed in one case, but this situation is described more frequently in the literature. It was reported in 20/51 vestibular neuritis patients by Li et al. [83] and was also described by Kobaya et al. [84].

Other publications [22,27] showed that symptom development was not correlated with objective test results (i.e., BCT, VHIT, SVINT) but could be linked to acquiring visual dependency [72] or perhaps due to central dysfunction, as described by Jauregui-Renaud et al. [85] and Staab et al. [86].

### 4.5. Limitations of This Study and Future Perspectives

Only a small number of patients in our study (group B) complained of persistent symptoms but had a normal set of vestibular tests. However, the literature reports higher percentages of patients with normalized laboratory tests but with persisting subjective symptoms [83]. The interpretation of this finding could be that the time interval between the onset of vestibular neuritis and that of the second assessment (three months) was not long enough for the development of PPPD in the B and C groups (of note is that the first criterion for PPPD diagnosis is the persistence of symptoms for at least three months or longer [87]).

VEMPs were not assessed for logistical reasons and because our interest was to focus on the semicircular canals portion of the vestibular apparatus. However, the SVINT is a global test that also stimulates the phasic receptors of semicircular canals and utricles [45,88].

The VHIT device used in our department did not provide the Perez-Rey index, so a latency pattern evaluation (gathered and scattered corrective saccades) was not possible. The Perez-Rey index was therefore replaced by a clinical assessment of the temporal dispersion of corrective saccades, which was not included in the study.

Further studies are scheduled that will analyze the vertical component of eye movements for spontaneous nystagmus and nystagmus evoked by the HST and SVINT. It is hoped that these studies will provide more information about the development of vestibular neuritis. Similarly, the performed Motion Sickness Susceptibility and Visual Vestibular Mismatch questionnaires will also be taken into account, in order to obtain a better understanding of patients who have totally recovered, versus those who have had to compensate for persisting pathology (as evidenced by abnormal vestibular laboratory tests).

## 5. Conclusions

The major conclusion of our study proposes that vascular risk factors (hypercholesterolemia) were correlated with patients presenting with persistent subjective symptoms and no recovery of objective vestibular tests.

Vestibular neuritis patients with an initial BCT hypofunction > 90% show no recovery of their BCTs and are left with a residual permanent lesion.

We propose a novel subdivision of vestibular neuritis patients into four groups, in order to evaluate and classify them more easily and provide follow-up for the development of the disease. This classification is based on the evaluation of subjective functional scale scores and the development of objective vestibular tests analyzing different frequencies and structures. This classification allowed us to uncover some patients with objective recovery as documented by testing, but who had persistent subjective symptoms corresponding to a diagnosis of possible PPPD.

We detected no significant difference in the development and recovery of high-frequency or low-frequency vestibular tests in patients who did not complain of subjective symptoms after three months. The HST was the test that recovered most often in 80% of the cases at T3. The HST recovery at 100% corresponds to the group of patients who have no longer any symptoms.

We suggest that patients detected at the acute period with hypercholesterolemia should be treated by the institution of statin therapy after cardiologic consultation and blood testing.

More information about the profiling of patients who recover by total recovery or compensation is expected from the complementary analysis of the vertical component of eye movements and the Motion Sickness Susceptibility and Visual Vestibular Mismatch questionnaires.

## Figures and Tables

**Table 1:** (a) Classification of patients based on the presence of normal or pathological laboratory tests, measured by the bithermal caloric test, video head impulse test, skull vibration-induced nystagmus test, and the presence or absence of subjective symptoms, measured by the Dizziness Handicap Inventory. For each of the four groups (A, B, C, D), the sample size is specified. DHI: Dizziness Handicap Inventory. A group (patients without symptoms and no vestibular laboratory test dysfunction); B group (patients with symptoms and no vestibular laboratory test dysfunction); C group (patients with symptoms and with vestibular laboratory test dysfunction); and D group (patients without symptoms and with vestibular laboratory test dysfunction). (b) The global result at T0 and T3 in the different groups concerning the Dizziness Handicap Inventory (DHI) is mentioned on the left side of the table. The number of patients (=n) for the bithermal caloric test (BCT), lateral semicircular canal video head impulse test (L VHIT), skull vibration-induced nystagmus test (SVINT), and head shaking test (HST) with positive vestibular tests at T0 and at T3 is mentioned on the right side of the table. A group (patients without symptoms and no vestibular laboratory test dysfunction); B group (patients with symptoms and no vestibular laboratory test dysfunction); C group (patients with symptoms and with vestibular laboratory test dysfunction); and D group (patients without symptoms and with vestibular laboratory test dysfunction). A + D group (patients without symptoms); B + C group (patients with symptoms); A + B group (patients with no vestibular laboratory test dysfunction); and C + D group (patients with vestibular laboratory test dysfunction).

(a)										
				**Without Symptoms** **DHI < 32**				**With Symptoms** **DHI ≥ 32**		
**No Vestibular Laboratory Test Dysfunction**				Total restitution → seven patients (17.5%)				Chronic dizziness → one patient (2.5%)		
				**A** group				**B** group		
**Vestibular Laboratory Test Dysfunction**				Subjective compensation but not objective recovery → 22 patients (55%)				Neither subjective compensation nor objective recovery → 10 patients (25%)		
				**D** group				**C** group		
		
(b)										
			**Positive Vestibular Tests (n=)**							
**Groups**	**DHI T0**	**DHI T3**	**L VHIT T0**	**L VHIT T3**	**SVINT T0**	**SVINT T3**	**HST T0**	**HST T3**	**BCT T0**	**BCT T3**
**A**	68.00 ± 5.29	16.00 ± 12.17	7	0	7	0	7	0	7	0
**B**	62	80	1	0	1	1	1	0	1	0
**C**	62.2 ± 11.25	61.00 ± 9.49	10	10	10	9	10	8	10	10
**D**	64.82 ± 15.52	18.36 ± 7.42	22	22	22	20	22	0	22	22
**A + D**	65.59 ± 13.73	17.79 ± 8.61	29	22	29	20	29	0	29	22
**B + C**	62.18 ± 10.68	62.73 ± 10.67	11	10	11	10	11	8	11	10
**A + B**	67.25 ± 5.34	24.00 ± 25.28	8	0	8	1	8	0	8	0
**C + D**	64.00 ± 14.20	50.00 ± 21.60	32	32	32	29	32	8	32	32

**Table 2:** Linear relationships presented as a correlation matrix including all patient results. For each pair of variables, the Pearson’s r value indicates the strength and direction of the relationship between two variables. A negative correlation indicates a variation in opposite sides of a pair of variables (for lateral semicircular canal VHIT VN side and BCT; for lateral semicircular canal VHIT VN side and SVINT; for anterior semicircular canal VHIT VN side and SVINT)—when the first variable increase, the second decrease. A positive correlation indicates a variation in the same side of a pair of variables (SVINT and BCT; anterior semicircular canal VHIT VN side; and lateral semicircular canal VHIT VN side)—when the first variable increases, the second also increases. Variables used include age, Dizziness Handicap Inventory (DHI), bithermal caloric test (BCT), number (Nb) of vascular risks, skull vibration-induced nystagmus test (SVINT) and video head impulse test for vestibular neuritis side for lateral (L VHIT), anterior (A VHIT), and posterior (P VHIT) semicircular canal. VN: vestibular neuritis.

		Age	DHI	BCT	Nb of Vascular Risks	SVINT	L VHIT VN Side	A VHIT VN Side	P VHIT VN Side
**Age**	Pearson’s r	—							
	*p* value	—							
**DHI**	Pearson’s r	−0.251	—						
	*p* value	0.188	—						
**BCT**	Pearson’s r	−0.017	−0.086	—					
	*p* value	0.931	0.656	—					
**Nb of Vascular Risks**	Pearson’s r	−0.112	0.322	0.284	—				
	*p* value	0.562	0.089	0.135	—				
**SVINT**	Pearson’s r	0.111	−0.008	**0.506** **	0.057	—			
	*p* value	0.566	0.968	0.005	0.769	—			
**L VHIT VN Side**	Pearson’s r	0.182	−0.250	**−0.716** ***	−0.298	**−0.623** ***	—		
	*p* value	0.345	0.191	<0.001	0.117	<0.001	—		
**A VHIT VN Side**	Pearson’s r	0.050	−0.141	−0.222	0.137	**−0.540** **	**0.481** **	—	
	*p* value	0.795	0.465	0.246	0.478	0.003	0.008	—	
**P VHIT VN Side**	Pearson’s r	0.178	−0.275	−0.233	−0.353	−0.169	0.304	0.364	—
	*p* value	0.356	0.148	0.224	0.060	0.381	0.109	0.052	—

** = *p* <0.01; ***= *p* <0.001; ns = *p* >0.05 (n = 29; Degree of Freedom = 27).

**Table 3:** Characterization of the four paired groups of patients concerning sex, age, and body mass index (BMI). A + D group (patients without symptoms); B + C group (patients with symptoms); A + B group (patients with no vestibular laboratory test dysfunction); C + D group (patients with vestibular laboratory test dysfunction).

Group	Sex	Age	BMI
**A + D**	15M/14F	65.10 ± 15.17	25.43 ± 3.42
**B + C**	6M/4F	70.37 ± 9.99	26.46 ± 4.05
**A + B**	4M/4F	63.89 ± 18.84	25.12 ± 3.54
**C + D**	17M/15F	67.22 ± 13.72	25.86 ± 3.64

**Table 4:** Historical comparison between the Eisenman et al. [56] and Guajardo-Vergara et al. [54] compensation criteria. It is necessary to consider the time interval between the proposals of these authors since, over twenty years, vestibular laboratory diagnostics have significantly evolved. Ny: nystagmus; SPV: slow phase velocity.

Eisenman et al. (2001) [56]	Guajardo-Vergara et al. (2020) [54]
Spontaneous Ny ≥ 2°/s	Videonystagmography: Spontaneous Ny ≥ 3°/s; direction-fixed positional Ny ≥ 4°/s in at least three of the four positions evaluated
Positional Ny persistent ≥ 3°/s in more than half of the eleven positions assessed, sporadic in nearly all positions, or ≥ 6°/s in any one position	Caloric test: Directional preponderance ≥ 25%
Directional preponderance ≥ 25%	Rotatory chair test: Sinusoidal Harmonic Acceleration Test: Asymmetry in the SPV ≥ 5°/s on at least three frequencies; Impulsive Test: Time Constant asymmetry < 20%
Asymmetry of slow-component eye velocity responses, outside of established normal ranges, at two or more of the six frequencies evaluated on rotatory chair testing	Clinical situation: Instability
	Dizziness Handicap Inventory: ≥ 32 total score

## Data Availability

Requests may be directed to Dr Enrico Armato (MD).

## Abbreviations

AVS: acute vestibular syndrome, AUVP: acute unilateral vestibulopathy, BCT: bithermal caloric test, BPPV: benign paroxysmal positional vertigo, CVRFs: cardiovascular risk factors, DHI: Dizziness Handicap Inventory, HINTS: head impulse, nystagmus, test skew, HST: head shaking test, NS: not significant, MRI: magnetic resonance imaging, PPPD: persistent postural–perceptual dizziness, SVIN: skull vibration-induced nystagmus, SVINT: skull vibration-induced nystagmus test, T0: first assessment at vestibular neuritis diagnosis time, T3: second assessment 90 ± 15 days (3 months) from the onset of vestibular neuritis symptoms, VEMPs: vestibular evoked myogenic potentials, VHIT: video head impulse test, VIF: variance inflation factor, VNG: videonystagmography, VOR: vestibulo-ocular reflex.
